# Lactoferrin could alleviate liver injury caused by Maillard reaction products with furan ring through regulating necroptosis pathway

**DOI:** 10.1002/fsn3.2254

**Published:** 2021-05-06

**Authors:** Linlin Fan, Fengen Wang, Qianqian Yao, Haoming Wu, Fang Wen, Jiaqi Wang, Huiying Li, Nan Zheng

**Affiliations:** ^1^ Key Laboratory of Quality & Safety Control for Milk and Dairy Products of Ministry of Agriculture and Rural Affairs Institute of Animal Science Chinese Academy of Agricultural Sciences Beijing China; ^2^ Laboratory of Quality and Safety Risk Assessment for Dairy Products of Ministry of Agriculture and Rural Affairs Institute of Animal Sciences Chinese Academy of Agricultural Sciences Beijing China; ^3^ State Key Laboratory of Animal Nutrition Institute of Animal Science Chinese Academy of Agricultural Sciences Beijing China

**Keywords:** 5‐hydroxymethylfurfural (5‐HMF), furosine, lactoferrin, Maillard reaction products (MRPs), pyralline

## Abstract

As classical MRPs, the toxic effects of furosine, pyralline, and 5‐hydroxymethylfurfural (5‐HMF) in liver tissue are evaluated and the related mechanism is investigated here, and the protective effects of lactoferrin on liver injury caused by Maillard reaction products (MRPs) with furan ring are proved in vitro and in vivo. First, we detect the concentrations of furosine, pyralline, and 5‐HMF in several foods using ultrahigh‐performance liquid chromatography (UHPLC). Then, the effects of the three MRPs on liver cells (HL‐7702) viability, as well as liver tissue, are performed and evaluated. Furthermore, the regulations of three MRPs on necroptosis‐related pathway in liver cells are investigated. Additionally, the effects of lactoferrin in alleviating liver injury, as well as regulating necroptosis pathway, were evaluated. Results elucidate that lactoferrin protects liver injury caused by MRPs with furan ring structure through activating RIPK1/RIPK3/p‐MLKL necroptosis pathway and downstream inflammatory reaction.

## Graphical abstract

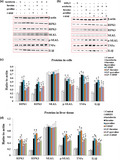

## 1 INTRODUCTION

The Maillard reaction (MR) is common during heat processing of food, especially dairy processing and baking. Diverse Maillard reaction products (MRPs) are generated during early, middle, and late stage of MR, including *N*(ε)‐2‐furoylmethyl‐l‐lysine (furosine), pyrraline, *N*(6)‐(1‐carboxyethyl)‐l‐lysine (CEL), fructolysine (FL), pronyl‐lysine, *N*(ε)‐carboxymethyl‐l‐lysine (CML), pentosidine, and 5‐hydroxymethylfurfural (5‐HMF; Henle, 2005; Huang et al., 2015; Ledl & Schleicher, 1990). MRPs can not only affect the sensory attributes of thermally processed foods, changing their appearance, flavor, aroma, and texture, but also damaging the nutritional value (Fan et al., 2018). Moreover, there are accumulating evidences showing that the consumption of MRPs might lead to multiple diseases, such as cataract, diabetes, degenerative diseases, atherosclerosis, and chronic renal failure. (Echavarría et al., 2012).

Among these MRPs, furosine (C_12_H_18_N_2_O_4_, Mw 254.28), pyralline (C_12_H_18_N_2_O_4_, Mw 254.29), and 5‐HMF (C_6_H_6_O_3_, Mw 126.11) share similar furan ring structure. As they are abundant in heating processed foods, the biological effects of them are attracting more attention in recent years. We have proved that furosine could pose toxic effects on liver and kidney tissue, as well as the reproductive system (Li, Xing, Wang, et al., 2018; Li, Xing, Zhang, et al., 2018; Li, Wang, Yang, Zhang, et al., 2019), and there are a few studies indicated that 5‐HMF might take adverse effects on human health, attributing to its induction of oxidation and inflammation (Nassberger, 1990). As previous researches showed that the exogenous MRPs could be absorbed into body and undergone complex metabolism in liver and kidney (Erbersdobler & Faist, 2001; Faist et al., 2001; Somoza et al., 2006), it is meaningful to take into account dietary heated foods, in which variable MRPs might exhibit potential deleterious effects on organs.

Lactoferrin, a nonheme iron‐binding glycoprotein (M.w. 80 KD), is a member in the family of transferrin. Lactoferrin is mainly secreted by mammary epithelium and highly existed in milk of different mammalian species. As a bioactive protein in foods, especially in dairy products, lactoferrin exerts multiple bioactive functions including iron metabolism, immunomodulation, anti‐inflammation, antioxidation, antibacteria, antivirus, and anticancer (Zhang et al., 2014). Referring to the protective effects of lactoferrin in liver injury, there were a few studies. Acetyl aminophenol‐induced liver injury (AILI) is a global health problem; previous research found that human lactoferrin could attenuate the degree of hepatohemia and damage of endothelial cells during AILI via suppressing macrophage (Prgomet et al., 2010; Yin, Cheng, Holt, et al., 2010). In a mouse model of hepatitis resulted from concanavalin (Con A), lactoferrin could alleviate inflammatory damage by suppressing the activation of T cells, the secretion of IFN‐α, and increasing the level of anti‐inflammatory factor IL‐4 (Yin, Cheng, Agarwal, et al., 2010). Bovine lactoferrin was proved to inhibit the concentration of proinflammatory factors (TNF‐α, IL‐6, NO) and stimulate the release of anti‐inflammatory cytokine (IL‐10) in serum, thus preventing acute liver failure of rat induced by GalN/LPS (Sugiyama et al., 2010). However, its effects on liver damages caused by MRPs were rarely reported.

Therefore, in the present study, we detected the contents of furosine, pyralline, and 5‐HMF in several foods, including milk powder, baked bread, coffee, and bee honey, to validate the abundance of the three MRPs in common food products. Thereafter, we constructed HL‐7702 cell (a normal hepatic cell line) model and ICR mouse model, and investigated the toxic effects of furosine, pyralline, and 5‐HMF on cell viability in vitro, as well as on liver function and liver pathological state in vivo. Additionally, the necroptosis pathway was focused according to our recent study (Li, Wang, Yang, Wang, et al., 2019; Li, Wang, Yang, Zhang, et al., 2019), to investigate the toxicity mechanism of MRPs with furan ring structure on liver tissue, and the expressions of its related proteins: RIPK‐1, RIPK‐3, and MLKL were verified by Western blotting. Especially, the protective effect of lactoferrin in alleviating liver injury caused by MRPs with furan ring was proved in vitro and in vivo. This study demonstrated that lactoferrin could protect liver damage caused by MRPs with furan ring structure, providing data basis for limit standards formulation of the three chemicals in foods, which presented necessity and urgency to a great extent.

## 2 MATERIALS AND METHODS

### 2.1 Chemicals

Furosine was purchased from PolyPeptide (Strasbourg, France), and the purity was greater than 95%. FL, CEL, CML, pyrraline, 5‐HMF, and lactoferrin were purchased from Sigma, with the purities of 95%. Human liver cell line (HL‐7702) was purchased from American Type Culture Collection (ATCC, Crofele). Roswell Park Memorial Institute 1,640 (RPMI 1,640) medium, fetal bovine serum (FBS), and 1% penicillin/streptomycin were purchased from GIBCO. Cell Counting Kit‐8 (CCK‐8) was purchased from Solarbio (Beijing, China). Determination kits of alanine transaminase (ALT), aspartate transaminase (AST), alkaline phosphatase (ALP), and γ‐glutamyl transferase (γ‐GT) in mice serum were purchased from Jiancheng (Nanjing, China). Hematoxylin–eosin (HE) staining kit was purchased from Solarbio. RIPA lysate buffer (including proteases and phenylmethylsulfonyl fluoride) and a protein concentration detection kit were obtained from Solarbio. The primary antibodies “recognizing receptor‐interacting serine/threonine‐protein kinase” (RIPK‐1, RIPK‐3), “mixed lineage kinase domain‐like protein (MLKL),” phosphorylated MLKL (p‐MLKL), and inflammatory factors TNF‐α and IL‐1β were purchased from Santa Cruz (San Francisco, USA). Additionally, the internal reference β‐actin, as well as the secondary antibodies, was also purchased Santa Cruz. Other reagents for Western blotting were purchased from Solarbio. Enhanced chemiluminescence (ECL) reagents were purchased from Tanon (Shanghai, China).

Acetonitrile (ACN), methanol, and trifluoroacetic acid (TFA) for sample preparation and liquid chromatography separation were obtained from JK Scientific (Beijing, China). Ammonium hydroxide, sodium hydroxide, hydrochloric acid (HCl), sulfuric acid, ammonia sulfate, and boric acid were purchased from Sinopharm Chemical Reagent (Beijing, China). Ultrapure water was prepared using a PURIST purification system (Lefeng, Shanghai, China). Pyrraline stock solutions (200 mg/L) were prepared in ultrapure water; 5‐HMF stock solutions (2 g/L) were prepared in methanol; and furosine stock solutions (500 mg/L) were prepared in 3 M HCl.

### 2.2 Cell culture and cell viability detection

HL‐7702 cells were cultured in RPMI 1,640 medium containing 10% FBS, 500 U penicillin/streptomycin, in an incubator (37℃, 5% CO_2_). The cells were implanted into 96‐well plates (10^4^ cells/well) and cultured for at least 24 hr, and the cells were exposed to furosine, FL, CEL, CML, pyrraline, HMF (0–1000 mg/L), and lactoferrin, respectively, and cocultured for 48 hr; then, cell viability was detected by CCK‐8 kit. Considering the principles of safety (viability > 80%) and effects (compared with the control, *p* < .05), the sensitive chemicals to HL‐7702 cells, as well as the proper dosages of lactoferrin and MRPs for further experiments, were chosen. Then, cell viabilities of the combinations of lactoferrin (pretreated with 2 hr) and sensitive MRPs were measured, to further observe the protection of lactoferrin.

### 2.3 Detection of furosine in bread, milk powder, coffee, and honey

One g sample of bee honey (Baihua, Beijing, China), 0.20 g sample of bread (baked by ourselves), milk powder (gathered from Holstein dairy cattles milk in our laboratory, processed by ourselves), or coffee (Nestle, Vevey, Switzerland) were mixed with 8 ml hydrochloric acid (8 mol/L) in a screw‐cap Pyrex1 tube with nitrogen exposure for 1 min, and then hydrolyzed at 110℃ for 23 hr with the tube tightly closed. After digestion, the supernatant was cooled to room temperature and filtered using filter paper (Jiaojie 101, Fushun, China). The supernatant was diluted six times with ultrapure water and filtered using 13 mm 0.20 µm GHP Minispike filters (Waters, Milford, MA, USA) for subsequent UPLC analysis.

Chromatographic separation was performed on a Waters Acquity UPLC system (Waters, Milford, MA, USA) equipped with a Waters 2,489 UV/VIS detector and an HSS T3 analytical C18 column (100 mm × 2.10 mm, 1.80 mm) in a column oven maintained at 37°C and an injection volume of 2 μl. A gradient program was applied, and the mobile phase consisted of combinations of solvent A (water), solvent B (water containing 0.1% trifluoroacetic acid), and solvent C (acetonitrile, Beijing Chemical Works) as follows: 45% A and 55% B (initial), 45% A and 55% B (0−0.5 min), 45−100% A (0.5−2.0 min), 0−100% C (2.0−2.1 min), and 100% C−45% A and 55% B (2.1−3.5 min). Re‐equilibration (4.5 min) was performed before the next injection. The flow rate was 0.4 ml/min, and the injection volume was 1.5 μL.

Total nitrogen content in samples for the final furosine calculations was determined using the Kjeldahl method as previously described (FIL/IDF 2001, Standard 20‐1) (International Organization for Standardization & International Dairy Federation, 2001).

The retention time of furosine was 2.12 min, with good separation from samples impurities. The limits of detection (LODs) were 0.10 mg/100 g in bee honey samples and 10 mg/100 g protein in bread, milk powder, and coffee samples according to recovery experiments. Furosine was quantified using a linear calibration curve built with furosine working solutions in the linear range 0.20 to 50.00 mg/L, with the coefficients of determination *R* ^2^ of .9999.

### 2.4 Detection of pyrraline in bread, milk powder, coffee, and honey

The sample was extracted by methanol (with 2% ammonium hydroxide) for 30 min using a KQ‐500B ultrasonator (45°C and 80 kHz, Kunshan, Jiangsu, China). The mixture was centrifuged (5,000 r/min) for 10 min. Five ml of the supernatant from bread or milk powder samples was dried by air, dissolved in 1 ml methanol/water (v/v, 1/9) solution, and filtered by 13 mm 0.45 µm GHP Membrance (Acrodisc® Syringe Filters, PALL, Port Washington, NY, USA) for subsequent HPLC analysis. For the supernatant from bee honey and coffee samples needed a purification by PEP column (Agela Technologies) before air drying, the PEP column was activated by methanol (3 ml) and washed by water for several times, and then added 1 ml supernatant, washed by water (2 ml), and eluted by methanol (3 ml). The purified supernatant was dried by air, dissolved in 1 ml methanol/water (v/v, 1/9) solution, and filtered by 13 mm 0.22 µm GHP Membrane (Acrodisc® Syringe Filters, PALL, Port Washington) for subsequent HPLC analysis.

Chromatographic separation was performed on a Waters 1525 HPLC system (Waters, Milford, MA, USA) equipped with a UV detector and an Agilent extend‐C18 column (250 mm × 4.6 mm, 5 µm). The injection volume was set at 20 μl. The mobile phase A was TFA 0.1% solution, and the mobile phase B was ACN/water (v/v, 1/1) solution. The analyte was eluted at 1.0 ml/min through the following gradient of solvent B (t [min]/[%B]): (0/0), (1/0), (10/15), (30/20), (35/100), (40/0), (45/0), and then detected at 297 nm.

The retention time of pyrraline was 13.10 min with good separation from impurities. The limit of detection (LOD) was 0.10 µg/g in bread, milk powder, bee honey, and coffee samples. Pyrraline was quantified using a linear calibration curve built with pyrraline working solutions in the linear range 0.0050 to 2.50 µg/L, with the coefficients of determination *R* ^2^ of .9999.

### 2.5 Detection of 5‐HMF in bread, milk powder, coffee, and honey

The sample was weighed in a tube after blending and extracted by methanol (with 2% ammonium hydroxide) for 30 min using a KQ‐500B ultrasonator (45℃ and 80 kHz, Kunshan, Jiangsu, China). The mixture was centrifuged (4℃, 10,000 r/min) for 10 min. Five mL of the supernatant from bread or milk powder samples was dried by nitrogen, dissolved in 1 ml methanol/water (v/v, 1/9) solution, and filtered by 13 mm 0.45 µm GHP Membrance (Acrodisc® Syringe Filters, PALL, Port Washington, NY, USA) for subsequent HPLC analysis. For the supernatant from bee honey and coffee samples needed a purification by C18 column (Agela Technologies) before nitrogen drying, the C18 column was activated by methanol (3 ml) and water (2 ml), and 2 ml supernatant was then added and washed by water (3 ml), and eluted by methanol/water (v/v, 4/1) (2 ml). The purified supernatant was dried by nitrogen, dissolved in 1 ml methanol/water (v/v, 1/9) solution, and filtered by 13 mm 0.22 µm GHP Membrance (Acrodisc® Syringe Filters, PALL, Port Washington, NY, USA) for subsequent HPLC analysis.

Chromatographic separation was performed on a LC‐20AT system (Shimadzu, Guangzhou, China) equipped with a UV detector and an Agilent extend‐C18 column (250 mm × 4.60 mm, 5 µm). The injection volume and column oven temperature were set at 20 μl and 30℃, respectively. The analyte was eluted at 0.80 ml/min through the isocratic methanol/water (v/v, 5/95) solution, and then detected at 280 nm.

The retention time of 5‐HMF was 8.40 min with good separation from impurities. The limit of detection (LOD) was 0.10 µg/g in bread, milk powder, bee honey, and coffee samples. 5‐HMF was quantified using a linear calibration curve built with 5‐HMF working solutions in the linear range 0.10 to 50.00 µg/ml, with the coefficients of determination *R* ^2^ of .9998.

### 2.6 Animal model

ICR mice were obtained from Beijing Vital River Laboratory Animal Technology Co., Ltd. with the license number SCXK 2012‐Animal experiments were approved by the Ethics Committee of Chinese Academy of Agriculture Sciences (Beijing, china) (Permission number: CAS20181015).

In acute toxicity model, 60 ICR mice (20 ± 2 g, female) were randomly divided into 12 groups (*n* = 5 per group): control group (without any treatment), ddH_2_O treatment group (0.20 ml/ mice, once every day), 0.10 g/kg b.w. furosine group, 0.25 g/kg b.w. furosine group, 0.50 g/kg b.w. furosine group; 0.10 g/kg b.w. pyralline group, 0.25 g/kg b.w. pyralline group, 0.50 g/kg b.w. pyralline group; 0.10 g/kg b.w. 5‐HMF group, 0.25 g/kg b.w. 5‐HMF group, 0.50 g/kg b.w. 5‐HMF group, and 0.25 g/kg b.w. lactoferrin group. The three MRPs and lactoferrin were prepared to 0.2 ml administration volume per mice. The mice were fasted 1 hr prior to dosing treatment, and the animals were observed individually after administration, for a total of 14 days for any clinical signs of toxicity or mortality. At the end of the 14th day, alive animals in acute toxicity model were sacrificed, and the proper dosages (without obvious symptoms) of the three chemicals were selected for further experiments.

In chronic toxicity model, 45 ICR mice (20 ± 2 g, female) were randomly divided into 9 groups (*n* = 5 per group): control group (without any treatment), ddH_2_O treatment group (0.20 ml/ mice, once every day), 0.25 g/kg b.w. lactoferrin group, 0.10 g/kg b.w. furosine group, 0.10 g/kg b.w. pyralline group, 0.10 g/kg b.w. 5‐HMF group, lactoferrin + furosine group, lactoferrin + pyralline group, and lactoferrin + 5‐HMF group. The mice in treatment groups were gavage administrated once per day (0.20 ml/ mice), in consecutive 21 days. The mice were sacrificed on the 22nd day, the blood sample was gathered from retro‐orbital plexus of mice eyes, and the liver tissue was dissected out, which should be frozen in liquid nitrogen for biochemical indicators detection and pathological staining.

### 2.7 Detection of biochemical indicators in mice serum and liver serum

The live tissue was cut into small pieces on ice, then homogenated by homogenate machine (ART Miccra, Germany), and the sample were gathered after centrifugation (1006 *g*, 10 min). The levels of TNFα and IL1β were measured. The mice blood was centrifuged to gather serum for biochemical indicators detection (15 min, 3,000 r/min at 4℃), including ALT, AST, ALP, and γ‐GT, which were detected by ELISA kits protocols (Jiancheng).

### 2.8 Necroptosis pathway detection in HL‐7702 cells and liver tissues by Western blotting

With 48‐hr treatments of lactoferrin, three MRPs, and the combinations, the HL‐7702 cells were gathered. And the total protein in cells and in liver tissue samples were extracted using a protein extraction kit (Solarbio) and measured by a BCA kit (Solarbio). After 10 min heat treatment of 95℃, the protein samples were added into the 10% SDS–polyacrylamide gels and the electrophoresis was performed, the proteins were transferred onto nitrocellulose membranes by Trans‐Blot machines (Tanon), and the filters were blocked with 2% BSA in TBST buffer for 1.5 hr at room temperature (RT). Then, the proteins were probed with different primary antibodies for 3 hr at RT, including β‐actin, RIPK‐1, RIPK‐3, MLKL, P‐MLKL, TNF‐α, and IL‐1β (Santa Cruz), respectively. The β‐actin was utilized to promise the equal loadings. After washing with PBST buffer (3 × 10 min), the membranes were incubated with the according secondary antibodies for 1.5 hr at RT and then washed with PBST buffer (4 × 15 min). Finally, the membranes were detected utilizing an ECL reagent and analyzed by Image J software (Rawak Software, Inc.).

### 2.9 Pathological staining of mice liver tissue

Liver tissue was isolated and fixed in 4% paraformaldehyde (Solarbio) for 24 hr before the tissues were embedded by paraffin and sliced up by slicing machine (Leica, Germany). The tissue sections were stained with hematoxylin and eosin (HE) for pathological observation, and examined under light microscope (Olympus, Japan). The proper photographs were taken as indicated (200× magnification).

### 2.10 Data analysis

All the data were represented as Mean ± *SD*. Data analysis was performed using GraphPad Prism 6.0 software (GraphPad, San Diego, USA). Statistical analysis was conducted using the Student's *t* test and one‐way analysis of variance (ANOVA). *p* value < .05 was considered to indicate a statistically significant difference between groups.

## 3 RESULTS

### 3.1 Furosine, 5‐HMF, and pyralline inhibited the viability of HL‐7702 cells

**FIGURE 1:**
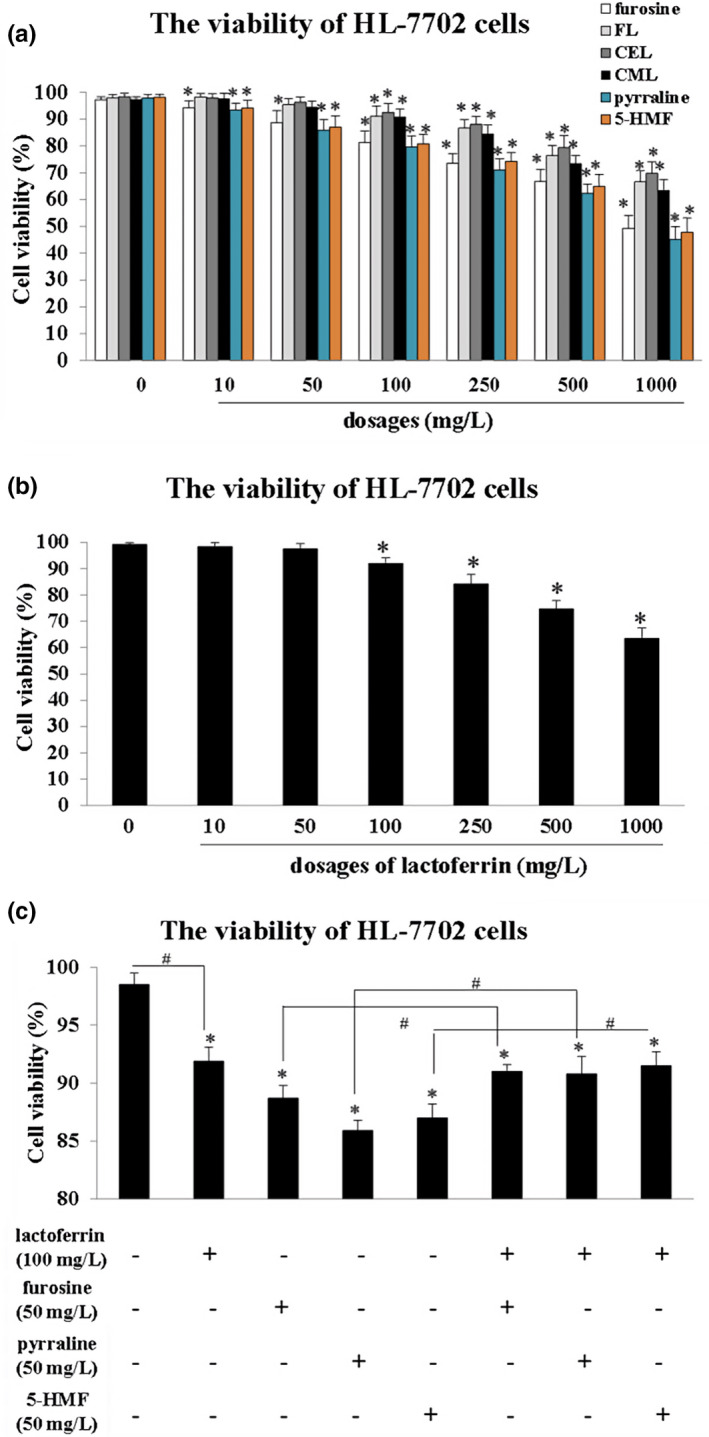
The viabilities of HL‐7702 cells detected by CCK‐8 kit. (a) Cell viabilities affected by furosine, FL, CEL, CML, pyralline, and 5‐HMF. (b) Cell viabilities affected by lactoferrin. (c) Cell viabilities affected by the combinations of lactoferrin and furosine/pyralline/5‐HMF. Data are presented as means ± *SD*, *n* = 8. *p < .05, compared with the control. #*p* < .05, compared with the single treatment groups

As Figure 1a demonstrated, six chemicals inhibited the viability of HL‐7702 cells in different degrees, and furosine, 5‐HMF, and pyralline could significantly inhibit cells survival rates in 10 mg/L group and 50 mg/L (compared with control, *p* < .05). However, the ones with the treatments of the other three chemicals (FL, CEL, CML) (10 mg/L group and 50 mg/L) seemed no obvious changes when compared with the control (Figure 1). Thus, 50 mg/L was selected as the proper dosage for furosine, 5‐HMF, and pyralline in further experiments.

### 3.2 Lactoferrin upregulated the viability of HL‐7702 cells when combined with furosine, 5‐HMF, or pyralline

Through CCK‐8 detection, dosages of 100 mg/L or above of lactoferrin could inhibit the viability of HL‐7702 cells significantly, when compared with the control (*p* < .05, Figure 1b). Thus, 100 mg/L was chosen as the proper dosage of lactoferrin. Furthermore, the cell viabilities of the combinations of lactoferrin (100 mg/L) + furosine/5‐HMF/pyralline (50 mg/L) were measured to observe the protection of lactoferrin in liver cells survival, and results demonstrated that with the pretreatment of lactoferrin, the cells viabilities in the combinations were significantly higher than the ones in single treatment groups (*p* < .05, Figure 1c), indicating that lactoferrin protected liver cells survival affected by MRPs with furan ring.

### 3.3 The contents detections of furosine, 5‐HMF, and pyralline in various foods

**FIGURE 2:**
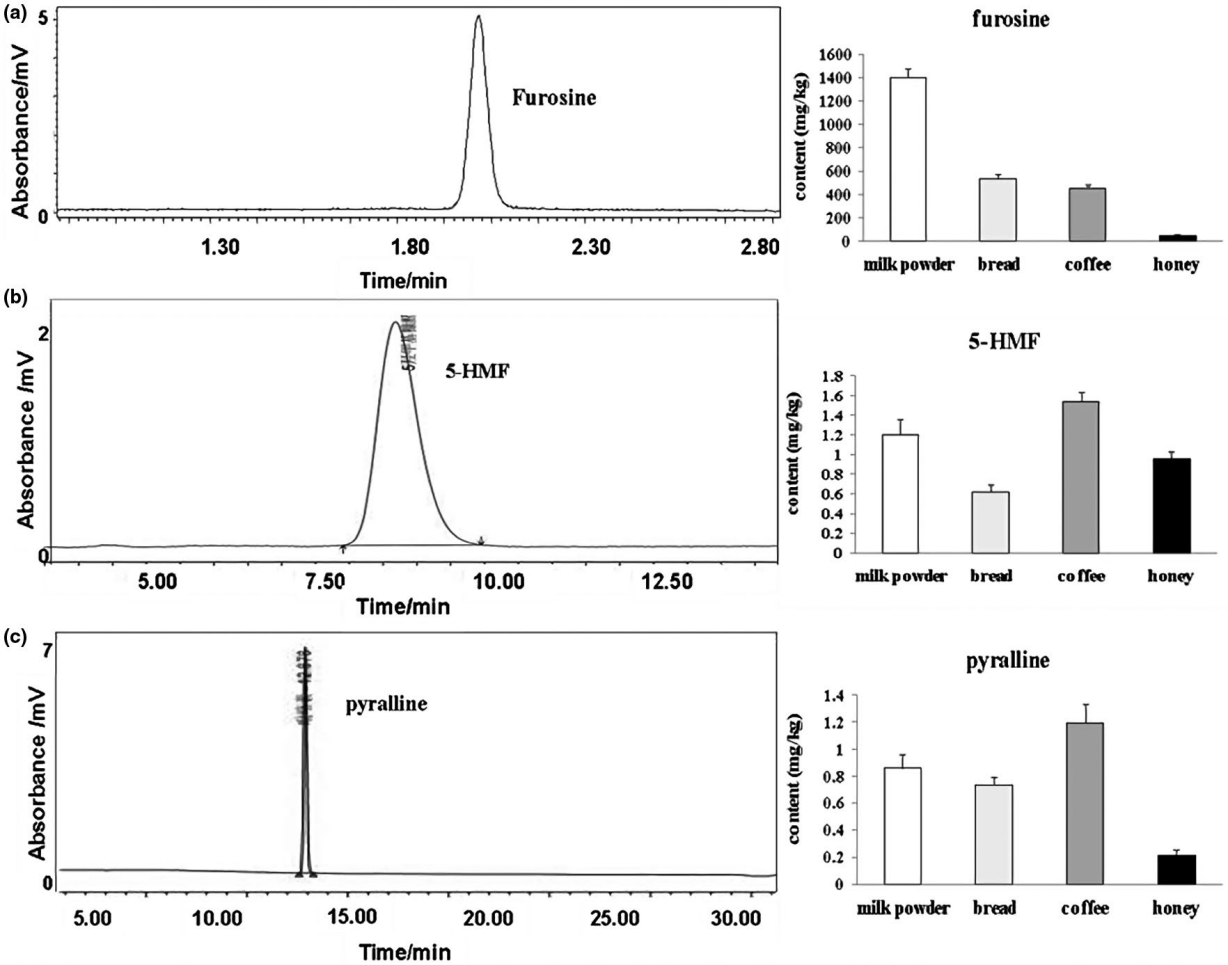
Detections of furosine, pyralline, and 5‐HMF in several heat processed food products. (a) Furosine in milk powder, baked bread, coffee, and bee honey. (b) 5‐HMF in milk powder, baked bread, coffee, and bee honey. (c) Pyralline in milk powder, baked bread, coffee, and bee honey. Data are presented as means ± *SD*, *n* = 3

In several extreme cases with excessive heating treatment, the content of furosine was 1,401.40 ± 76.70 mg/kg in milk powder, 533.50 ± 41.20 mg/kg in baked bread, 455.10 ± 28.40 mg/kg in coffee, and 52.50 ± 8.00 mg/kg in bee honey. Typical chromatogram of sample containing furosine is shown in Figure 2a. The content of 5‐HMF in milk powder samples was 1.20 ± 0.15 mg/kg, and 0.62 ± 0.079 mg/kg in bread, 1.54 ± 0.093 mg/kg in coffee, and 0.96 ± 0.073 mg/kg in bee honey (Figure 2b). The content of pyralline in milk powder samples was 0.86 ± 0.097 mg/kg, and 0.74 ± 0.057 mg/kg in bread, 1.20 ± 0.14 mg/kg in coffee, and 0.22 ± 0.038 mg/kg in bee honey (Figure 2c).

### 3.4 Dosages selection in animal model

In acute toxicity model, single oral administration of furosine, pyralline, and 5‐HMF with three dosages were performed, and the physical status of mice was observed for 14 days. Slight anorexia and diarrhea were observed 2–4 days later in groups of 0.25 g/kg b.w. of the three chemicals. Symptoms such as hypoactivity and asthenia occurred with 14 days after the oral administration of 0.50 g/kg b.w. of the three chemicals. Thus, 0.10 g/kg b.w. was selected as the proper dosage of furosine, pyralline, and 5‐HMF, in further chronic toxicity model.

### 3.5 Biochemical indicators detections in mice serum and histopathologic detection of liver tissue treated with lactoferrin, three MRPs, and their combinations

**FIGURE 3:**
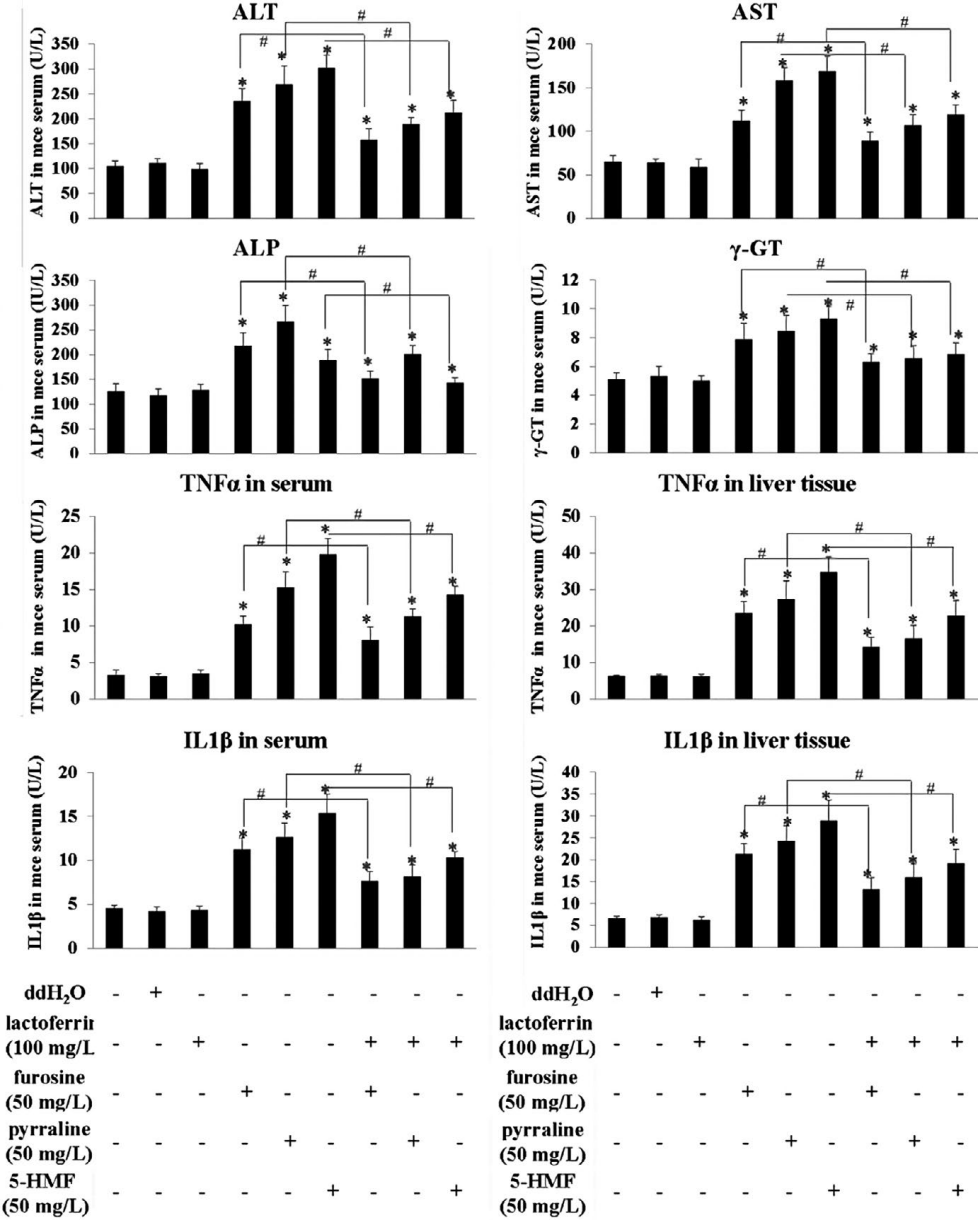
The effects of lactoferrin, three MRPs, and their combinations on biochemical indicators (ALT, AST, ALP, γ‐GT, TNF‐α, and IL‐1β) in mouse serum and liver tissue, measured by ELISA kits. Data are presented as means ± *SD*, *n* = 5. *p < .05, compared with the control. #*p* < .05, compared with the single MRPs treatment groups

**FIGURE 4:**
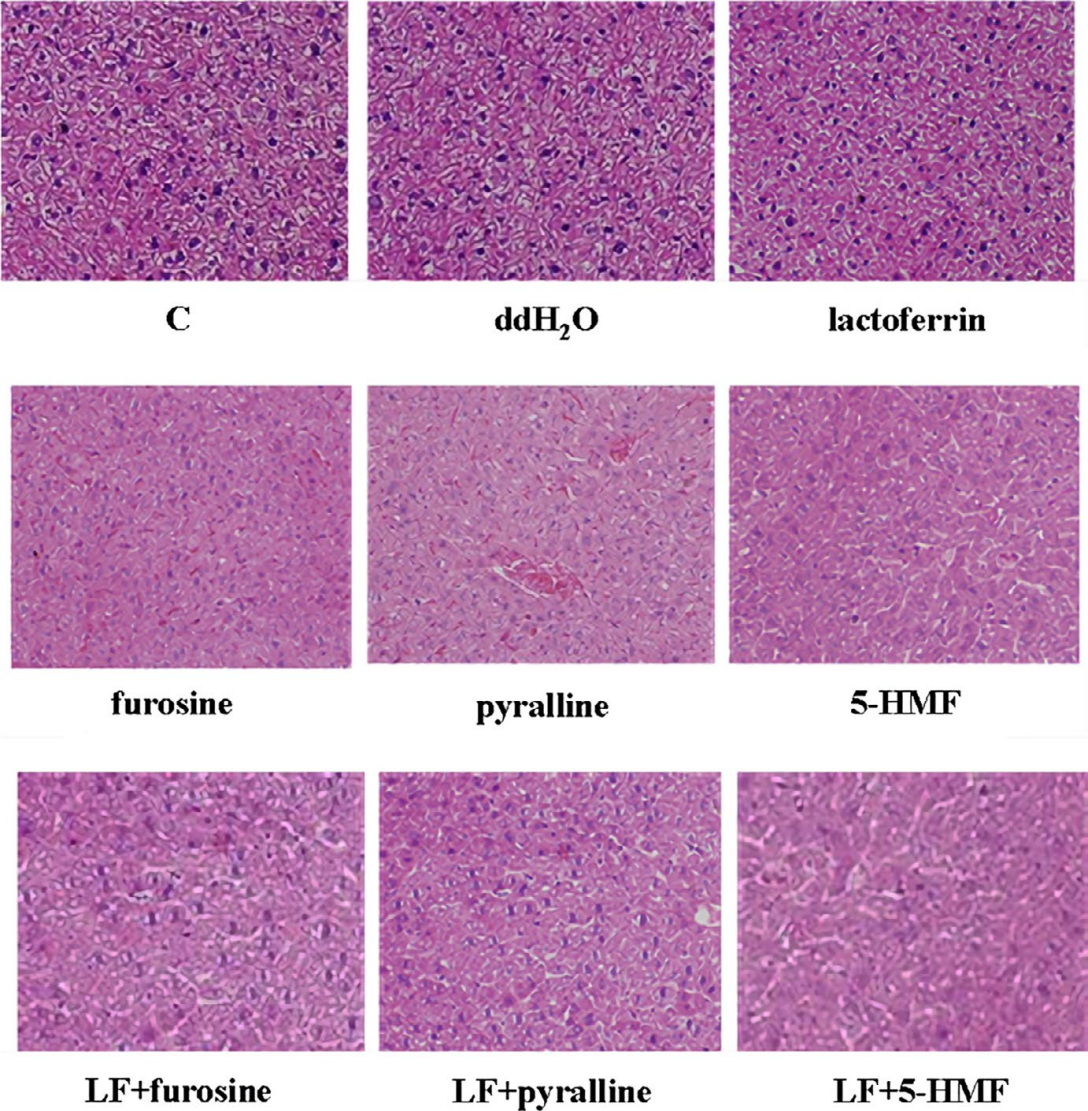
Pathological detection of mouse liver tissue by HE staining. Under 200× magnification

A 22‐day consecutive administration of furosine, pyralline, and 5‐HMF at a dosage of 0.10 g/kg b.w. significantly upregulated the content of AST, ALT, ALP, γ‐GT, TNF‐α, and IL‐1β in mice serum and liver tissue, in comparison with the control (*p* < .05), and their levels in the combinations were significantly lower than the ones in furosine/pyralline/5‐HMF groups (*p* < .05, Figure 3). Further HE staining of liver tissue showed that the three MRPs at a dosage of 0.10 g/kg b.w. caused occasional cytomorphosis and slight edema in some areas in the liver tissue, and lactoferrin showed protective effects in these damages (Figure 4). Both of the blood biochemistry tests and histopathologic assays proved that furosine, pyralline, and 5‐HMF indeed caused liver damage in vivo, and lactoferrin could alleviate these liver damages in some degrees.

### 3.6 Furosine, 5‐HMF, and pyralline caused hepatocytes injury through activating necroptosis pathway, in both hepatocytes and liver tissue

**FIGURE 5:**
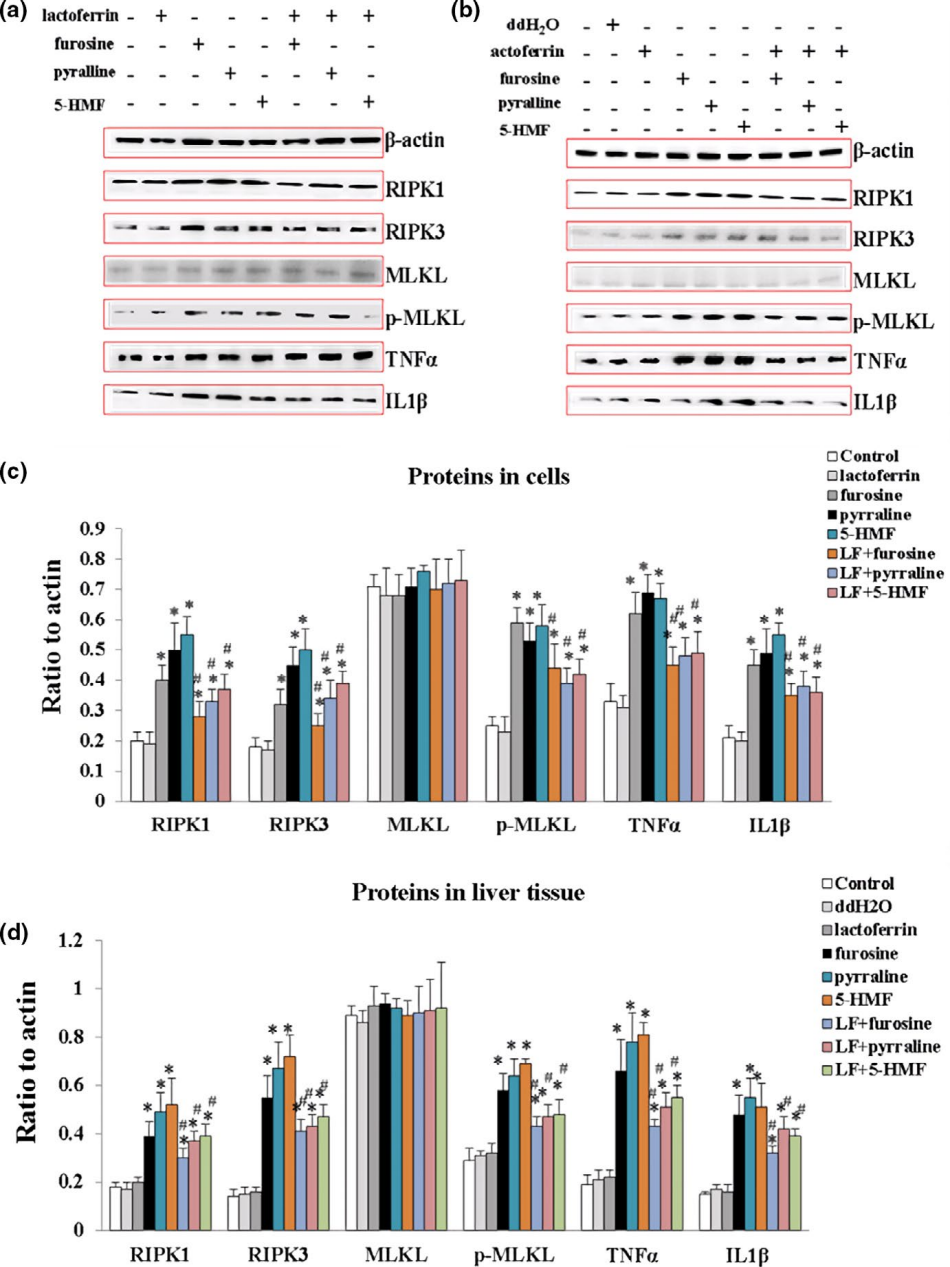
The regulations of lactoferrin, three MRPs, and their combinations on RIPK1, RIPK3, MLKL, p‐MLKL, TNF‐α, and IL‐1β by Western blotting. (a) Protein bands in HL‐7702 cells. (b) Protein bands in mouse liver tissue. (c) Quantification of proteins in HL‐7702 cells. (d) Quantification of proteins in mouse liver tissue. Data are presented as means ± *SD*, *n* = 3. * *p* < .05, compared with control. #*p* <.05, compared with the single MRP treatment groups

In our previous study, we found that furosine could trigger necroptosis in primer hepatocytes by regulating RIPK1/RIPK3/MLKL pathway [6]. In this study, to further detect the effects of three chemicals on necroptosis pathway in HL‐7702 cells and mice liver tissue, the expressions of RIPK1, RIPK3, MLKL, and p‐MLKL, as well as inflammatory factors TNF‐α and IL‐1β, were detected by Western blotting. Results demonstrated that RIPK1, RIPK3, p‐MLKL, TNF‐α, and IL‐1β both in hepatic cells and in liver tissue were significantly upregulated, when compared with the control (*p* < .05), and with the combination of lactoferrin, the expression levels of these proteins were downregulated significantly when compared with the MRPs treatment groups (*p* < .05, Figure 5), suggesting furosine, 5‐HMF, and pyralline caused liver damage partly through activating necroptosis pathway.

## 4 DISCUSSION AND CONCLUSION

The quantification analysis of furosine, 5‐HMF, and pyralline in milk powder, baked bread, coffee, and bee honey proved the abundance of them in various heating processed foods (Liska et al., 2015). In our latest research, furosine was proved to cause liver and kidney injury through inducing downstream inflammation and excessive apoptosis of normal cells (Li et al., 2018), furosine could also evoke reproductive toxicity through regulating CEP55‐related pathway in male mouse primary spermatogoniums (Li, Wang, Yang, Wang, et al., 2019; Zhang et al.,^1993^ *),* and proved that 5‐HMF might act as an initiator as well as a promoter in the induction of colon cancer in mice model, through upregulating the growth of aberrant crypt foci (ACF) (Zhang et al., 1993). 5‐HMF has also been found to possess mutagenic and DNA strand‐breaking activity (Rosin et al., 1982). However, the toxicity evaluation and mechanism research of pyralline are rarely seen. Due to lack of experimental data about overall toxicity of the three chemicals, the standards for tolerable daily intake (TDI) and their limit standards in foods, especially furosine and pyralline, have not been established. The present manuscript aimed to validate the toxic effects of furosine, pyralline, and 5‐HMF on liver, as well as to investigate the particular relationship between their toxicity mechanism and furan ring structure.

Interestingly, we observed that all of the three chemicals have the furan ring, which may be responsible for their similar physiological toxicity. Furan was proven to have toxicity to Fischer‐344 rat, and the NOAEL (no‐observed‐adverse‐effect level) of furan was as low as 0.03 mg/kg b.w. per day. Changes in clinical biochemistry and hematological parameters were observed at a dose of > 0.5 mg/kg b.w. (Gill et al., 2010). Repeated oral administration of furan could induce mild histological alterations with necrosis, apoptosis, and limited regenerative cell proliferation to B6C3F1 mice. Besides, furan at high dosage brought about a significant increase of DNA damage in mouse liver (Cordelli et al., 2010). Thus, we guessed that furan ring in the MRPs might also be a potential site exerting toxic effects. In this study, we found that HL‐7702 cells were more sensitive to furosine, pyralline, and 5‐HMF, when compared with other three MRPs (FL, CEL, CML), further indicating furan ring structure played a key role in the toxicity of liver cells. In mouse toxicity model, by measuring several biochemical indicators related with liver functions, we found that the levels of ALT, AST, ALP, and γ‐GT were upregulated with the treatments of furosine, pyralline, and 5‐HMF. For these biochemical indicators reflected the condition of organ functions, abnormal expressions of ALT, AST, ALP, and γ‐GT in mice serum proved that the MRPs with furan ring affected liver functions and resulted in hepatic injury. Moreover, the inflammatory factors (TNF‐α and IL‐1β) in serum and liver tissue were also measured, which further indicated MRPs with furan ring could activate necroptosis in liver tissue, and lactoferrin significantly alleviates inflammation caused by MRPs with furan ring. Combined with the pathological results of HE staining, we guessed that furosine, pyralline, and 5‐HMF might accumulate and metabolize in liver tissue, which embodied in inducing cytomorphosis and blood vessel rupture, evoking inflammation and tissue edema. Therefore, we proved that liver was at least one of major target organs of MRPs with furan ring structure.

As a newly discovered pathway in the latest decade, necroptosis of programmed cell necrosis requires the proteins RIPK1, RIPK3, and RIPK3 substrate MLKL, which can be activated by interferons, death receptors, intracellular RNA and DNA sensors, etc. (Pasparakis & Vandenabeele, 2015). In this course, in response to the activation of the above factors, RIPK1 is recruited to the cytosolic side of the receptor, and then, its kinase activity is activated. RIPK1 interacts with a related kinase, RIPK3, leading to its activation (Degterev et al., 2008; He et al., 2009), RIPK3 subsequently phosphorylates a pseudokinase called MLKL, which normally exists as an inactive monomer in the cytosol (Liao et al., 2017). Once MLKL is activated and translocates to cell plasma membrane, it could disrupt membrane integrity and prompt the release of inflammatory factors, like TNF‐α and IL‐1β, finally leading to cell necrosis (Chen et al., 2014; Hildebrand et al., 2014; Rodriguez et al., 2015; Wang et al., 2014). Necroptosis played a key role in several kinds of diseases, including sclerosis, cardiac reperfusion injury, enteritis, and organ injury, especially liver injury (Yeh et al., 2019), embodying on the increased expression levels of RIPK1, RIPK3, and p‐MLKL (Gookin et al., 2018; Liao et al., 2016; Pasparakis & Vandenabeele, 2015; Saeed & Jun, 2014; Scholz & Eder, 2017). Thus, the present study detected expressions of necroptosis proteins and inflammatory proteins to prove the regulation of furosine, pyralline, and 5‐HMF on liver cells necroptosis, further to explore the toxicity mechanism of MRPs with furan ring in liver tissue. Additionally, the roles of lactoferrin in regulating RIPK1/RIPK3/MLKL necroptosis pathway were detected, to identify whether lactoferrin could protect liver damages caused by MRPs with furan ring through regulating necroptosis.

To conclude, we proved that furosine, pyralline, and 5‐HMF increased the expression levels of RIPK1, RIPK3, p‐MLKL, TNF‐α, and IL‐1β significantly, validating that MRPs with furan ring activated necroptosis pathway and downstream inflammation in liver tissue. More interestingly, as a special bioactive protein in foods, lactoferrin could alleviate liver damages caused by MRPs with furan ring through inhibiting RIPK1/RIPK3/MLKL necroptosis pathway. A solid research of the effects of MRPs with furan ring on health, not only for its relation to diseases, but also for their toxic mechanism, could prompt researchers making efforts on optimizing food heating process to minimize the formation content of MRPs with furan ring, as well as to maintain safety of food products. Additionally, our data might also improve our understanding of risk assessment of MRPs with furan ring in foods and provide corresponding guidelines for limit standard, which presented necessity and urgency to a great extent.

## CONFLICT OF INTEREST

The authors declare that they do not have any conflict of interest.

## ETHICAL STATEMENT

Ethical Review: The animal experiment was approved by the Ethics Committee of Chinese Academy of Agriculture Sciences (Beijing, china) (Permission number: CAS20181015).
